# A novel experimental model of orthopedic trauma with acute kidney injury in obese Zucker rats

**DOI:** 10.1002/phy2.97

**Published:** 2013-10-02

**Authors:** Peter N Mittwede, Lusha Xiang, Silu Lu, John S Clemmer, Robert L Hester

**Affiliations:** Department of Physiology and Biophysics, Center for Excellence in Cardiovascular-Renal Research, University of Mississippi Medical CenterJackson, Mississippi

**Keywords:** Acute kidney injury, glomerular filtration rate, obese, orthopedic, trauma

## Abstract

Obesity is associated with an increased risk of acute kidney injury (AKI) after blunt traumatic injury in humans. Because limitations exist in studying trauma in human patients, animal models are necessary to elucidate mechanisms of remote organ injury after trauma. We developed a model of severe orthopedic trauma in lean (LZ) and obese (OZ) Zucker rats, in which OZ develop greater kidney dysfunction after trauma than LZ. Orthopedic trauma was inflicted via bilateral hindlimb soft tissue injury, fibula fracture, and injection of homogenized bone components. Mean arterial pressure (MAP) and heart rate (HR) were measured for 6 h after trauma, and again at 24 h after trauma. Urine was collected for 24 h before and after trauma to measure urine albumin excretion. Glomerular filtration rate (GFR), renal plasma flow (RPF), plasma interleukin-6 (IL-6), and renal macrophage infiltration (ED-1 [CD68 Antibody] immunostaining) were measured in animals with and without trauma. MAP and HR were similar between LZ and OZ throughout the study, with the exception that OZ had a 18 mmHg lower pressure 24 h posttrauma. GFR and RPF were decreased significantly (∼50%), while urine albumin excretion, plasma IL-6, and renal ED-1-positive cells were increased in OZ 24 h after trauma compared to both OZ without trauma and LZ after trauma. In conclusion, these data are consistent with studies in humans that show that AKI develops more frequently in obese than in lean individuals. This model will be an important experimental tool to better understand the underlying mechanisms of poor outcomes after trauma in obese patients.

## Introduction

The prevalence of obesity in adults in the United States is currently estimated to be over 35% (Flegal et al. 2012), and this trend of weight gain is expected to continue. In addition to increasing the risk of developing a number of different chronic disorders, including hypertension, diabetes, and cardiovascular disease (Calle et al. 1999), it is increasingly recognized that obese patients experience worse outcomes following trauma. This includes a greater risk for severe medical complications, including acute kidney injury (AKI) (Shashaty et al. 2012), acute respiratory distress syndrome (Belzberg et al. 2007), and multiple organ failure (Ciesla et al. 2006; Newell et al. 2007; Liu et al. 2012), all of which lead to significantly higher healthcare costs (Russell et al. 2011) and longer intensive care unit lengths of stay (Ciesla et al. 2006).

Acute kidney injury is a particularly devastating outcome after trauma, and its general mechanisms have been studied (Bonventre and Yang 2011; Bellomo et al. 2012). The development of AKI is associated with a greatly increased mortality rate in critically ill patients (Uchino et al. 2005). However, the reasons why it occurs with greater frequency in obese patients are not known. Our understanding of the pathophysiological mechanisms that increase the risk of AKI in obese patients after trauma has been significantly impeded by the lack of suitable experimental models.

The obese Zucker rat (OZ) is a model of metabolic syndrome that shares many similarities with humans who have this condition, including obesity, dyslipidemia, insulin resistance, and hypertriglyceridemia (Stepp et al. 2004). In this study, we sought to determine if orthopedic trauma in OZ would result in systemic and renal hemodynamic dysfunction consistent with AKI in humans.

## Material and Methods

### Animals

Male lean Zucker rats (LZ) and OZ (12–13 weeks old) were acquired from Harlan Laboratories (Indianapolis, IN). The animals were housed 2–3 per cage at 22°C, exposed to a 12-h light/dark cycle, and fed standard rat chow (Teklad, Harlan Laboratories). All protocols were approved by the Institutional Animal Care and Use Committee at the University of Mississippi Medical Center, and followed the guidelines set forth in the National Institutes of Health's *Guide for the Care and Use of Laboratory Animals*.

### Experimental groups

Four general experimental animal groups were used in this study (as labeled in the figures): OZ-Control and LZ-Control (with no trauma), and OZ-Trauma and LZ-Trauma (24 h after trauma). As described below, certain measurements (mean arterial pressure [MAP] and heart rate [HR]) were made prior to trauma, during the several hours following the trauma, and again at 24 h after trauma.

### Orthopedic trauma model

During 2 min of anesthesia via isoflurane inhalation, orthopedic trauma was induced in LZ and OZ. The trauma was based on a protocol previously described by our laboratory, with modifications made to make the injury more severe (Xiang et al. 2010). Briefly, the soft tissue behind the femur and tibia of each hindlimb was compressed with Mixter hemostatic forceps for 30 sec, followed by rapidly injecting 1.5 mL of a homogenized bone component suspension (2-gr crushed bone in 5 mL of phosphate buffered saline) bilaterally into the region of the injured muscles. The bones used to make the bone component suspension to inject into LZ and OZ were taken from previously sacrificed LZ and OZ, respectively. The fibula was fractured in each leg during the injection of the bone components. Directly before the trauma, the rats were given a subcutaneous injection of buprenorphine (0.01 mg/kg) to minimize discomfort, and every 8–12 h after trauma, they were given an additional dose of 0.05 mg/kg. The rats were monitored carefully after trauma with hemodynamic and temperature measurements being taken. This trauma model was used to simulate a bilateral femur fracture, but without the necessity of internal or external fixation surgery (fibula fracture does not necessitate this surgery), which has been shown to be a “second hit” that can lead to multiple organ failure and increased mortality (Waydhas et al. 1996; Keel and Trentz 2005). The soft tissue damage is similar to what would occur with a severe long bone fracture, and the crushed bone suspension mimics the marrow and other bone components that would be present following a fracture. This model was developed in order to investigate the effects of orthopedic trauma alone, and for this reason there were no treatment efforts.

### Systemic hemodynamics

At least 4 h prior to the orthopedic trauma the rats were anesthetized with inhalation of isoflurane (4–6%) and oxygen (100%), and polyethylene catheters (Intramedic PE-50 tubing; BD, Franklin Lakes, NJ) were surgically inserted into the left internal jugular vein and the right common carotid artery following a midline incision. The venous catheter was used for the infusion of substances to measure renal function (as described below), and the arterial catheter was used to monitor MAP and HR using PowerLab software (Model ML118; ADInstruments, Colorado Springs, CO). Following the catheter surgeries, a small dose of Sensorcaine (APP Pharmaceuticals, Schaumburg, IL) was injected locally to provide analgesia and reduce stress from the procedure. The animals woke up quickly and did not show signs of stress following these operations. MAP and HR were measured continually for 6 h following trauma, and again at 24 h after trauma.

### Renal hemodynamics

To determine glomerular filtration rate (GFR) and renal plasma flow (RPF) in LZ and OZ rats in the control and trauma groups, tritiated inulin (3H) (Perkin Elmer Health Sciences, Shelton, CT) and para-aminohippuric acid (PAH) (Sigma-Aldrich, St. Louis, MO) were mixed in 0.9% saline and a bolus of 0.2 mL of this solution was infused into rats via their jugular vein catheters. The solution was infused at 0.5 mL/h for 2 h to reach a steady-state level in the plasma, and then blood samples (<0.15 mL) were drawn at 15-min intervals and centrifuged at 1500 rpm for 10 min. The plasma concentration of 3H was determined via gamma counter (LS-6500; Beckman Coulter, Brea, CA). To calculate GFR and RPF, standard clearance formulas were used. The PAH concentration was measured with a spectrophotometer (Genesis 10UV; Thermo Spectronic, Rochester, NY). Both GFR and RPF were normalized by kidney weight.

### Plasma glucose and insulin

Fasting blood glucose levels (following a 4- to 6-h fast) were acquired in LZ and OZ from tail venous blood using a glucometer (On Call Plus; Acon Laboratories, San Diego, CA). Plasma insulin levels were measured using an ELISA kit (Crystal Chem, Inc., Downers Grove, IL).

### Urine albumin excretion

Urine was collected from a separate group of LZ and OZ by placing them in metabolic cages (Lab Products Inc., Seaford, DE) for 24 h prior to the administration of trauma, and then again for 24 h directly following trauma. Urine albumin concentration was determined with a Nephrat II ELISA kit (Exocell, Philadelphia, PA), and this value was multiplied by urine volume to get the total urine albumin excretion for 24 h.

### Plasma interleukin-6 and renin activity

Plasma interleukin-6 (IL-6) levels have been shown to be a marker of the systemic inflammatory response, as well as a good prognostic indicator for outcomes following traumatic injury (Frink et al. 2009). In a separate group of animals, blood was collected from LZ and OZ with no trauma and from those animals which had trauma 24 h earlier. The blood was centrifuged, and the plasma samples were used to measure IL-6 levels by ELISA (R&D Systems, Minneapolis, MN), and plasma renin activity (PRA) by radioimmunoassay (Perkin Elmer Health Sciences, Waltham, MA).

### Renal immunohistochemistry

The end point of the study was 24 h after trauma, at which time the animals were euthanized with an overdose of isoflurane, followed by bilateral pneumothorax. Mid-hilar kidney cross sections were quickly collected and fixed in formalin. These kidney sections were then stained with ED-1 (anti-CD 68) antibody (Abcam, Cambridge, MA), a monoclonal macrophage marker, as an index of renal inflammation. At least 20 glomeruli per kidney slice were randomly selected, and the number of ED-1-positive cells per glomerular cross section was counted by the first author of the study (P. N. M.).

### Statistical analysis

Data were analyzed with SPSS statistical software (Version 19; IBM, New York, NY), with groups being compared with student's *t*-tests or two-way analysis of variance (ANOVA), as appropriate. Where significant effects were found, Holm–Sidak post hoc tests were performed. All data are presented as mean ± SEM and *P* < 0.05 was considered statistically significant.

## Results

### Baseline characteristics

OZ had a significantly higher weight, fasting plasma glucose, and plasma insulin compared to LZ, while resting HR and MAP levels were not statistically different (Table 1).

**Table 1:** Baseline characteristics of obese and lean Zucker rats

Animal group	OZ	LZ
Weights	455 ± 5*	330 ± 7
Baseline mean arterial pressure	125 ± 4	118 ± 3
Resting heart rate	370 ± 7	370 ± 5
Fasting plasma glucose	116 ± 4*	96 ± 2
Plasma insulin	9.4 ± 1.1*	1.8 ± 0.2

Values are presented as mean ± SEM; *n* = 8 for OZ (obese Zucker rats), *n* = 8 for LZ (lean Zucker rats).

* *P* < 0.05 versus LZ.

### Renal hemodynamics

**Figure 1:**
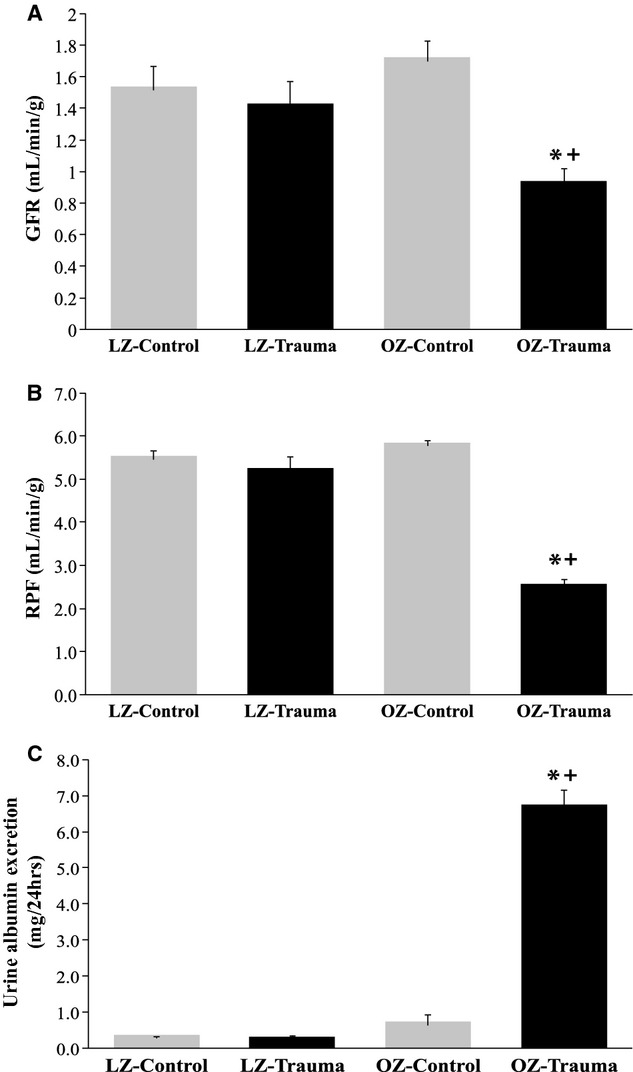
Glomerular filtration rate (GFR) and renal plasma flow (RPF) are decreased at 24 h after trauma in obese rats, while urine albumin excretion is increased. GFR was measured using the inulin plasma clearance method in LZ- and OZ-Control animals and in those which had trauma. RPF was measured with the plasma clearance method using para-aminohippuric acid (PAH). In LZ, neither GFR (A) nor RPF (B) was different between control and trauma animals. However, in OZ, GFR and RPF decreased significantly after trauma. LZ rats showed no difference in total albumin excretion (C) after trauma as compared to pretrauma. OZ albumin excretion was significantly increased after trauma (OZ-Trauma) as compared to pretrauma (OZ-Control). *n* = 6 or more for all groups. *P < 0.05 versus OZ-Control, +*P* < 0.05 versus LZ-Trauma.

GFR was not different between LZ- and OZ-Control (Fig. 1A). However, GFR was significantly lower in OZ rats 24 h after trauma, while GFR in the LZ was not different between trauma and control animals. Similarly, RPF was lower in OZ rats 24 h after trauma (Fig. 1B) but was not different between LZ- and OZ-Control.

### Urine albumin concentration

There was no change in urine albumin excretion in pre- versus posttrauma LZ (labeled as control and trauma in Fig. 1C). However, in OZ, urine albumin excretion increased significantly after trauma as compared to before trauma (*P* < 0.05).

### Systemic hemodynamics

**Figure 2:**
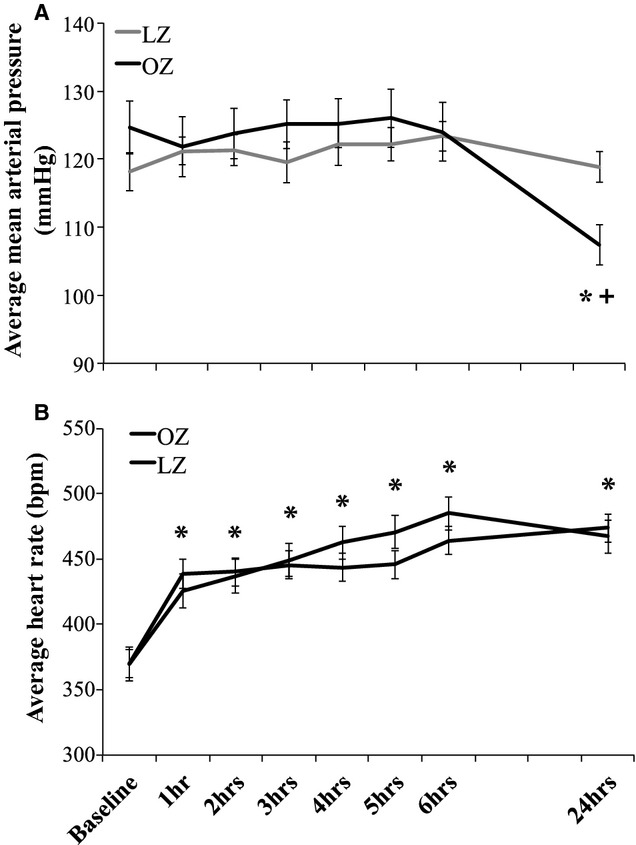
Mean arterial pressure (MAP) and heart rate (HR) responses for six consecutive hours after trauma, and at 24 h after trauma. Average MAP (A) and HR (B) were measured via carotid catheters for six consecutive hours after trauma in LZ and OZ, and again at 24 h after trauma. There was no significant MAP change from baseline in either the LZ or OZ group for 6 h after trauma, but the day after trauma the MAP of OZ had decreased significantly both compared to baseline OZ levels, and to LZ-Trauma. HR gradually increased over a period of 6 h after trauma, and remained high at 24 h after trauma in both groups of animals. There were no significant differences between LZ and OZ animals at any time points. *n* = 8 (OZ), 6 (LZ). *P < 0.05 versus baseline, +*P* < 0.05 versus LZ at 24 h.

Figure 2A shows that the average MAP was unchanged in LZ or OZ for 6 h following trauma (with no acute hypotensive episodes noted). However, at 24 h after trauma, MAP in the OZ had decreased significantly (from 125 ± 4 at baseline to 107 ± 3 mmHg), while MAP in the LZ was not different from baseline. Average HR in LZ and OZ increased after trauma (Fig. 2B), and remained significantly higher than baseline the day after trauma in both LZ (474 ± 17 bpm) and OZ (467 ± 5 bpm), with no differences seen between groups at any time point.

### Plasma renin activity

**Figure 3:**
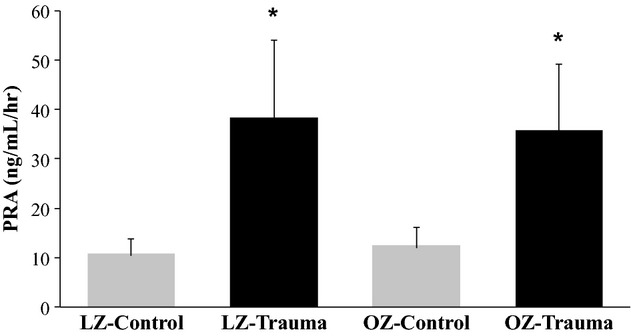
Plasma renin activity (PRA) is increased similarly in obese and lean rats 24 h after trauma. PRA was measured via radioimmunoassay in control and trauma LZ and OZ. Both the LZ and OZ PRA was significantly increased after trauma as compared to control values, and no differences were seen between LZ-Trauma and OZ-Trauma animals. *n* = 6 for all groups. *P < 0.05 versus OZ- and LZ-Control.

Plasma renin activity increased significantly in both LZ and OZ after trauma as compared to levels in control animals (Fig. 3). However, there was no significant difference in the PRA of LZ and OZ after trauma.

### Systemic inflammation

**Figure 4:**
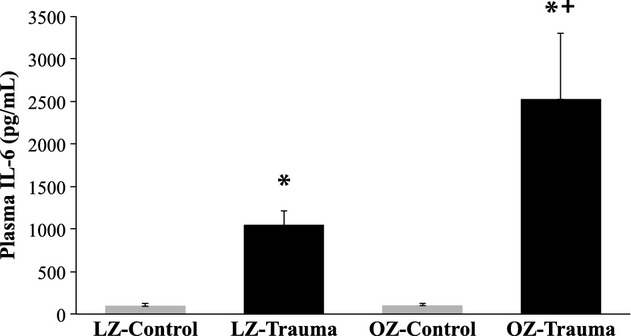
Systemic inflammatory response to trauma is more severe in obese than in lean rats. Plasma IL-6 levels (measured via ELISA) were increased significantly in both LZ- and OZ-Trauma animals as compared to control animals. OZ-Trauma rats had IL-6 levels that were significantly higher than in LZ-Trauma rats. *n* = 6 for all groups. *P < 0.05 versus Control, +*P* < 0.05 versus LZ-Trauma.

In control LZ and OZ there was no difference between plasma IL-6 levels (Fig. 4). In LZ 24 h after trauma, IL-6 levels were significantly higher than in control LZ. In OZ after trauma, levels were significantly higher than both LZ after trauma and control OZ.

### Renal inflammation

**Figure 5:**
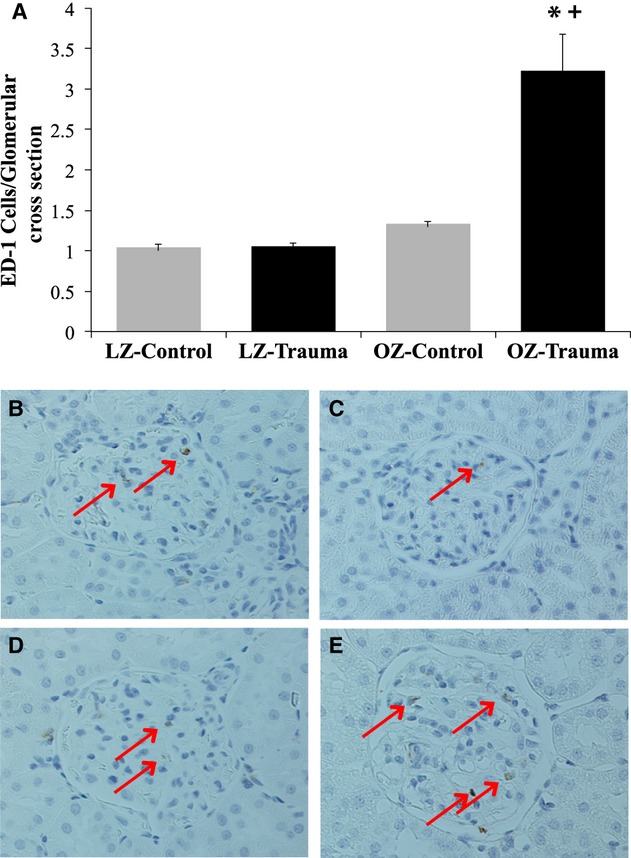
Renal (ED-1-positive cells) inflammatory response to trauma is more severe in obese than in lean rats. ED-1-positive cells (marker of macrophage infiltration) were counted in 20 randomly selected glomeruli in mid-hilar kidney sections from control and trauma LZ and OZ. No differences in ED-1-positive cell numbers were seen between control and trauma LZ, while the numbers were significantly increased in OZ after trauma (A). Representative histological sections can be seen for LZ-Control (B), LZ-Trauma (C), OZ-Control (D), and OZ-Trauma (E) animals. *n* = 5 for all groups. *P < 0.05 versus Control, +*P* < 0.05 versus LZ-Trauma.

ED-1-positive macrophage numbers were not different in the glomeruli of LZ after trauma as compared to control LZ (Fig. 5A). However, OZ after trauma showed significantly increased ED-1-positive cell numbers in their glomeruli (around a threefold increase) as compared to control OZ. The relative ratios of ED-1-positive cell numbers in the tubulointerstitial regions between groups were similar to those seen in the glomeruli (data not shown). Representative histological sections can be seen (Fig. 5B–E).

## Discussion

The overall purpose of this study was to test the hypothesis that orthopedic trauma in the OZ rat would lead to impaired renal function that is consistent with AKI in humans. The major new findings of our study are as follows: (1) orthopedic trauma results in AKI within 24 h in OZ with no effect in LZ, (2) OZ exhibit increased systemic and renal inflammatory responses to trauma as compared to LZ, and (3) OZ and LZ have a similar hemodynamic response to trauma initially, but OZ show, on average, a 18 mmHg decrease in MAP 1 day after trauma.

Clinical trauma and critical care literature show that obese patients develop a number of complications more frequently than their lean counterparts following traumatic injury. These include AKI, acute respiratory distress syndrome, and other complications (Liu et al. 2012). In this study, we focused on developing a model of trauma-induced AKI because its incidence after trauma is associated with a greatly increased mortality rate as well as larger costs to patients (Brandt et al. 2007). Between lean and obese trauma patients, mortality rates are controversial (Brown et al. 2005; Duane et al. 2006; Hoffmann et al. 2012), but a recent meta-analysis by Liu et al. 2012 found that, overall, obese individuals have an increased mortality rate after trauma as compared to those who are lean.

Trauma models have been developed to study posttraumatic responses in a number of animal species, including dogs, pigs, primates, mice, and rats (Tsukamoto and Pape 2009). However, our model is novel not only in its comparison of obese and lean rodents, but also in its nature of being a nonhemorrhagic simulation of blunt trauma in rats. Many blunt traumatic injuries seen in critical care settings after motor vehicle collisions or falls do not have a hemorrhagic component to them, and our model is meant to simulate these types of injuries.

The development of AKI is multifactorial, and the general pathophysiological mechanisms have been studied (Bonventre and Yang 2011; Bellomo et al. 2012). Overall, AKI occurs in around 26% of blunt trauma patients (Bihorac et al. 2010). However, the underlying mechanisms contributing to the increased risk of developing AKI following trauma in obesity are not clear. In the clinical setting, kidney function is typically evaluated with circulating creatinine and urea concentrations, as well as urine output. This gives clinicians inadequate information about structural and functional aspects of AKI, and it is often difficult to pinpoint the precise cause. Thus, animal studies are needed to help provide a deeper understanding of the impaired posttraumatic renal function in obese as compared to nonobese patients. Our model is one in which obese rats develop AKI after trauma, while lean rats do not. Characterizing this model, and the kidney injury that occurs, is an important first step in determining the mechanisms responsible for the deleterious outcomes seen in the obese animals.

In our study, the OZ had a significantly decreased RPF and GFR the day after trauma. Urine albumin excretion has been shown to be an early biomarker for intrinsic AKI (Ware et al. 2011), and the OZ exhibited increased excretion after trauma, suggesting development of AKI. PRA, a marker of renin–angiotensin system activity, was also increased significantly in both the LZ and OZ after trauma, with no differences between LZ and OZ. Increased renal sympathetic nerve activity can directly stimulate the juxtaglomerular cells of the kidney to secrete renin (DiBona and Kopp 1997). Via intrarenal vasoconstriction, activation of the renin–angiotensin system can lead to decreased GFR and RPF. However, the LZ did not have a decrease in GFR or RPF, suggesting that despite having a large increase in PRA, these animals may have compensatory mechanisms that preserve their kidney function. Possibilities include an upregulation of vasodilators or a downregulation of vasoconstrictors of the renal vasculature. In the OZ, these mechanisms either may not be present or may not be functioning properly. HR increased significantly in both the LZ and the OZ for 6 h after trauma and remained high the day after trauma (no differences between LZ and OZ at any time point), possibly due to increased sympathetic nervous system (SNS) activity. Increases in SNS activity can potentially lead to decreases in GFR and RPF, but as the GFR and RPF were intact in LZ, this is unlikely to fully account for the impaired renal function in the OZ.

Neither the OZ nor the LZ had a significant rise or fall in MAP in the few hours after trauma, but in the OZ, MAP had decreased 15 mmHg from baseline by the next day. The exact cause of this decrease in MAP is not clear, nor is its contribution to the decreased RPF and GFR. Renal autoregulatory mechanisms normally keep renal perfusion pressures stable across a wide range of systemic blood pressure levels, and a decrease in MAP of around 15 mmHg is unlikely to cause a 50% decrease in RPF and GFR, even if autoregulation is impaired.

We chose to study kidney function at the 24-h mark following trauma. One reason is that the development of AKI in this early time period after trauma is common (Bagshaw et al. 2008; Bihorac et al. 2010) and is associated with increased in-hospital mortality. Another reason is that our orthopedic trauma model is severe, and many of our animals do not survive 48 h posttrauma. Although there is no definitive evidence that AKI is the direct cause of death either in trauma patients or in our rat model, it is likely a contributing factor – AKI has been shown to predict multiple organ failure in trauma patients (Wohlauer et al. 2012), likely via the emerging concept of organ crosstalk (Doi et al. 2011).

OZ exhibited both an increased systemic (plasma IL-6) and renal inflammatory response the day after trauma as compared to LZ. IL-6 is a commonly used marker of systemic inflammation and it has also been shown to be an excellent prognostic indicator for outcomes after trauma (Lausevic et al. 2008; Frink et al. 2009). Following severe traumatic injury, an imbalanced inflammatory response has been implicated in the development of multiple organ failure (Dewar et al. 1993; Keel and Trentz 2005). The initial systemic inflammatory response to trauma is followed by a compensatory anti-inflammatory response, which has the potential to lead to immune suppression and subsequent infections (Keel and Trentz 2005). Our model, however, deals with the early response to trauma when systemic inflammation is still greatly elevated, particularly in the OZ.

Inflammation has been shown to be a culprit in both the initial development and progression of AKI. Accumulation of inflammatory cells in the kidney can lead to tubular and endothelial cell damage, as well as intrarenal vasoconstriction (Akcay et al. 2009). Decrements in kidney function can then stimulate the inflammatory response further and lead to subsequent distant organ damage (Ahuja et al. 2012; Bihorac et al. 2013). Thus, the exacerbated systemic and renal inflammation seen in the OZ may be either a cause or a consequence of the AKI, or both.

There were limitations to our study. Traumatic injuries that occur to humans are heterogeneous, and it is not possible to generalize all trauma to one type or to precisely simulate any particular type of human injury in animals. Nevertheless, we have attempted to create a model of severe orthopedic trauma with which we can study and attempt to understand mechanisms of posttraumatic remote organ failure. We believe that kidney injury after trauma is a complex phenomenon with many contributing factors, and it is highly unlikely that a single treatment will prevent the development of this disorder in our model. This study lays the groundwork for future studies we will undertake to further understand the disparate posttraumatic physiological and pathophysiological responses seen in obesity.

### Perspectives and Significance

Obese individuals develop AKI more frequently than their lean counterparts following traumatic injury, but the mechanisms of this have not been elucidated. In the current study, we have characterized a model of severe orthopedic trauma in rats, in which OZ develop AKI within 1 day, while LZ do not. This target organ damage after trauma occurs in the absence of hemorrhagic shock, but in association with increased systemic and renal inflammation in OZ as compared to LZ. The contribution of inflammation in accounting for the differences in renal function after trauma in LZ and OZ, as well as in humans, requires further study. This model will help us gain a better understanding of the causes underlying the increased incidence of AKI development in obese patients after trauma.
